# Effects of platelet-rich fibrin on osteogenic differentiation of Schneiderian membrane derived mesenchymal stem cells and bone formation in maxillary sinus

**DOI:** 10.1186/s12964-022-00844-0

**Published:** 2022-06-15

**Authors:** Jia Wang, Yue Sun, Yiping Liu, Jize Yu, Xiaolin Sun, Lin Wang, Yanmin Zhou

**Affiliations:** 1 Department of Oral Implantology, Hospital of Stomatology, Jilin University, Changchun, 130021 China; 2 Stomatology Hospital, School of Stomatology, Zhejiang University School of Medicine, Zhejiang Provincial Clinical Research Center for Oral Diseases, Key Laboratory of Oral Biomedical Research of Zhejiang Province, Cancer Center of Zhejiang University, Hangzhou, 310006 China

**Keywords:** Schneiderian membrane, Mesenchymal stem cells, Platelet rich fibrin, Osteogenic differentiation

## Abstract

**Background:**

The existence of mesenchymal stem cells (MSCs) in Schneiderian membrane has not been determined. The aim of this study is to investigate whether there are MSCs in Schneiderian membrane, and the effect of platelet-rich fibrin (PRF) on osteogenic differentiation of these cells and on new bone formation in maxillary sinus after maxillary sinus floor elevation.

**Methods:**

Schneiderian membrane derived mesenchymal stem cells (SM-MSCs) were isolated from rabbit maxillary sinus. Cells were identified by flow cytometry and multipotential differentiation. Real-time cell analysis assay, fluorescence staining, transwell assay, and wound healing assay were used to determine the effects of PRF stimulation on cell proliferation and migration. The osteogenic differentiation ability of cells stimulated by PRF or osteoinductive medium was evaluated by alkaline phosphatase staining, alizarin red staining, PCR and Western blot. Equivalent volume Bio-oss and the mixture of Bio-oss and PRF were used as bone graft materials for maxillary sinus floor elevation. Micro-CT, bone double-staining, HE staining, Masson staining, and toluidine blue staining were used to evaluate the osteogenic effect in 8 and 12 weeks after surgery.

**Results:**

The cell surface markers were positive for expression of CD90, CD105, and negative for expression of CD34, CD45. SM-MSCs had the ability of osteogenic, adipogenic and chondrogenic differentiation. PRF could stimulate proliferation, migration and osteogenic differentiation of SM-MSCs, which was achieved by up-regulating ERK 1/2 signaling pathway. PRF could accelerate the formation of new bone in maxillary sinus and increase the amount of new bone formation.

**Conclusions:**

MSCs existed in Schneiderian membrane, and PRF stimulation could promote cell proliferation, migration and osteogenic differentiation. The application of PRF in maxillary sinus floor elevation could accelerate bone healing and increase the quantity and quality of new bone. PRF, as autologous graft materials, might offer a promising strategy for the clinical bone formation during MSFE procedure.

**Graphical abstract:**

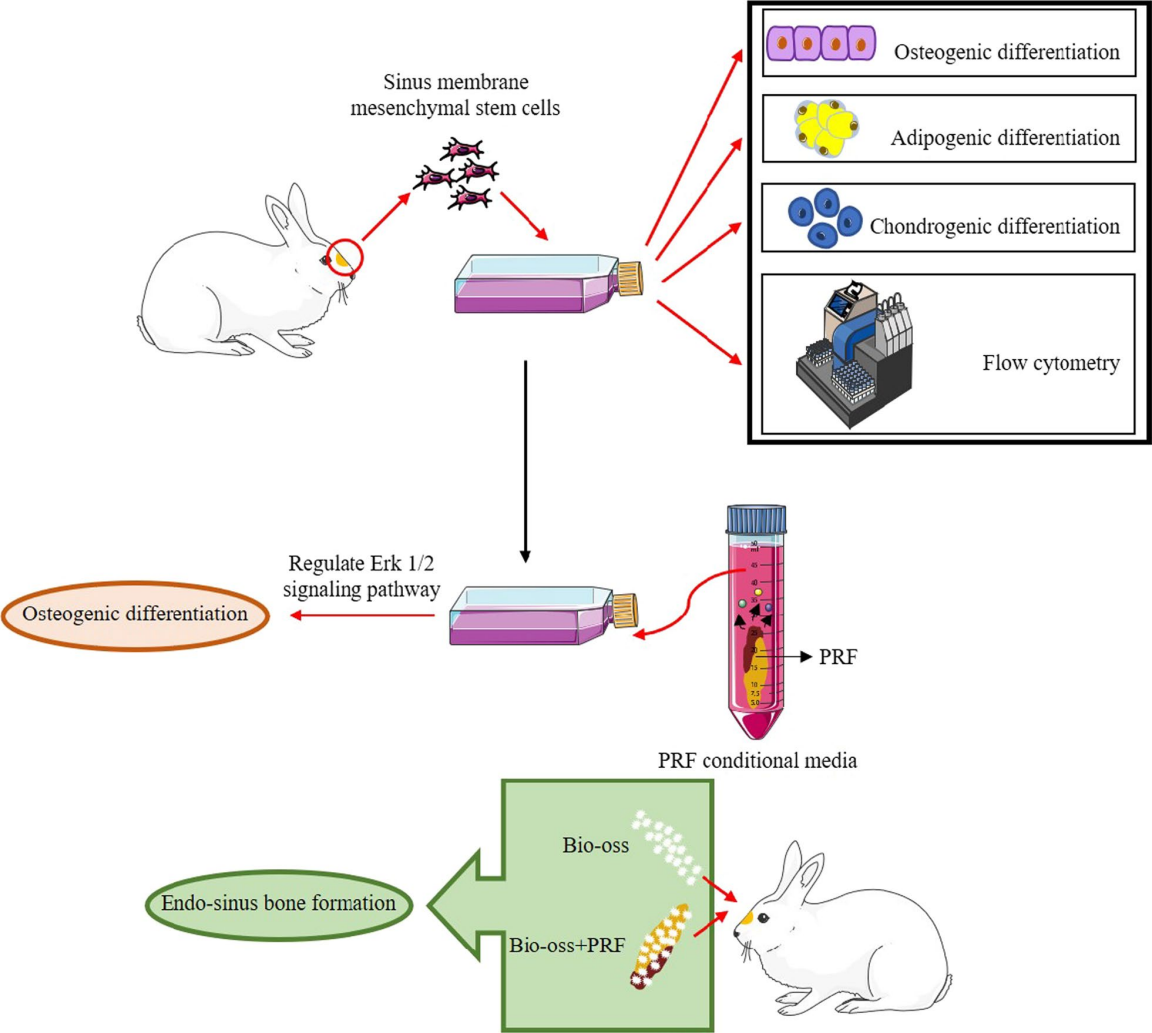

**Video Abstract**.

**Supplementary Information:**

The online version contains supplementary material available at 10.1186/s12964-022-00844-0.

## Background

Inadequate alveolar bone height is a common problem for dental implant placement in posterior maxilla. Maxillary sinus floor elevation (MSFE) had been reported as a procedure for resolving the inadequate bone height in the posterior maxilla [1, 2]. The principle of maxillary sinus floor elevation is to surgically peel and elevate the Schneiderian membrane from the maxillary sinus floor, with or without bone graft materials to increase the available bone height. The presence of the osteoblast is crucial in bone formation between Schneiderian membrane and maxillary sinus floor. Some scholars believed that mesenchymal stem cells (MSCs), migrated from the exposed alveolar bone for bone formation [3]. Whereas, other studies have proposed that the Schneiderian membrane may also contribute to the newly formed bone [4]. Some clinical studies have also demonstrated that the bone formation in the augmented area could be achieved by MSFE without bone graft materials [4]. Lundgren et al*.* and Jung et al*.* respectively found the spontaneous bone formation near the Schneiderian membrane after the teeth extraction or cyst removal in the maxillary sinus [5, 6], suggesting the presence of cells with osteogenic potential in the Schneiderian membrane. Therefore, many studies have been devoted to exploring the presence of MSCs in Schneiderian membrane. Gruber et al*.* had firstly reported that Schneiderian membrane contained mesenchymal progenitor cells and the cells could increase their osteogenic differentiation via responding to Bone Morphogenetic Protein-6 (BMP-6) and BMP-7 [7]. These findings were also confirmed by Berbéri [8]. In addition, Yun et al*.* found that the Schneiderian membrane derived MSCs could be induced by simvastatin [9]. Guo et al*.* confirmed that Schneiderian membrane derived MSCs could be induced to differentiate into a variety of lineages [10].

Recently, the current methods for osteogenesis induction on Schneiderian membrane derived MSCs are mainly the addition of exogenous reagents [11, 12]. However, even BMP-2, the only clinically approved osteogenic inductive growth factor, has been reported to exert numerous side effects such as ectopic bone formation, osteoclast-mediated bone resorption and inappropriate adipogenesis when the concentration is not appropriate [13]. Moreover, osteogenic differentiation of MSCs in vivo is carried out under complex conditions not a single inductive condition [14]. Platelet rich fibrin (PRF), a platelet concentrate proposed by Choukroun, contains many autologous growth factors, promoting cell proliferation and differentiation [15]. In recent years, many studies have reported that PRF could promote osteogenic differentiation of MSCs from different tissue sources. In our previous study, it was found that PRF enhances osteogenic lineage differentiation of alveolar bone progenitors by augmenting osteoblast differentiation, RUNX2 expression, and mineralized nodule formation [16]. Nugraha also confirmed the expression of early markers of osteogenic differentiation in gingiva MSCs stimulated by PRF was increased [17]. In another study, PRF stimulation promoted osteogenic differentiation and calcium-nodular mineralization of periodontal ligament stem cells compared with non-PRF stimulation [18]. Our previous clinical studies have found that using PRF alone as bone graft material in MSFE could obtain a certain amount of new endo-sinus bone [19], which is consistent with the results of Cho and Molemans et al. [20, 21] Thus, we hypothesized the presence of PRF may contribute to the bone regeneration in MSFE procedure. However, the precise biological behaviors and its possible mechanism that PRF efficiency on the differentiation of Schneiderian membrane derived MSCs are either unexplored.

In the present study, MSCs in Schneiderian membrane were successfully isolated and identified in vitro. Then, the proliferation, migration and osteogenic differentiate of Schneiderian membrane (SM-MSCs) with PRF were confirmed in vitro, and the underlying mechanisms was further explored. In vivo study, PRF could enhanced the formation of new bone in maxillary sinus. Taken together, PRF, as autologous graft materials, might offer a promising strategy for the clinical bone formation during MSFE procedure.

## Materials and methods

### Isolation and culture of SM-MSCs

SM-MSCs was separated from the maxillary sinus of 10 New Zealand white rabbits (aged 3 months, and weighing 3–3.5 kg). The present study was approved by the Institutional Animal Care and Use Committee of Jilin University (SY202008003). Rabbits were anesthetized with 0.1% pentobarbital sodium (3 mL/kg) through intramuscular injection. Local anesthesia was induced by 0.5 mL of 1% lidocaine hydrochloride to reduce bleeding and to provide analgesia at the surgical site. A vertical incision was made at the middle of the nasal dorsum to expose nasal bone and the sagittal suture line. Two rectangular windows (5 mm × 10 mm) were made using a round bur at both nasal bones and cooling with sterile saline solution. Windows were located about 20 mm anterior to the nasofrontal suture line and 4 mm away from the sagittal suture line. Schneiderian membrane was separated and stored in tubes containing saline solution. After rinsed with PBS, Schneiderian membrane was digested in a solution of 3 mg/mL collagenase type I (Sigma, USA) and 4 mg/mL dispase (Roche, Germany) for 1 h at 37 °C to remove the epithelium. The remaining tissue and cells are collected by centrifugation and seeded into 25 cm^2^ culture dishes with α-MEM (Hyclone, USA), supplemented with 10% fetal bovine serum (FBS, Gibco, USA), 100 U/mL penicillin and streptomycin (P/S, Hyclone, USA), and then incubated at 37 °C in 5% CO_2_. The medium had been changed every three days and the third to fifth passage cells were used in subsequent experiments.

### Flow cytometry

Approximately 1 × 10^6^ SM-MSCs were suspended in 1 mL PBS after washed three times and incubated with 10 μg primary antibody (FITC anti-CD34, Thermo Fisher scientific, USA; FITC anti-CD45, Bio-Rad, UK; FITC anti-CD90, BD biosciences, USA; FITC anti-CD105, Genetex, USA; FITC anti-IgG 1, Biolegend, USA) for 1 h in dark on ice. The negative control was incubated with PBS. Flow cytometry analyses were performed on a flow cytometer (BD Biosciences, USA).

### Osteogenic differentiation

The SM-MSCs were seeded at a density of 1 × 10^4^/well in 24-well plates in complete culture medium until confluent. For osteogenic differentiation, cells were cultured with osteoinductive medium (OM, α-MEM medium containing 10% FBS, 1% P/S, 10 mM β-glycerophosphate (Sigma, USA), 50 μg/mL ascorbic acid (Sigma, USA) and 10 nM dexamethasone (Sigma, USA)). The control group was cultured with complete medium (α-MEM medium containing 10% FBS, 1% P/S). Cells were cultured by replacing medium every three days. After culturing for 7 days, 14 days 21 days, cells were fixed in 4% paraformaldehyde and respectively stained with BCIP/NBT Alkaline Phosphatase Color Development Kit (Beyotime, China) and Alizarin Red S solution (1%, pH 4.2, Solarbio, China). The alkaline phosphatase (ALP) activity assay was followed the manufacturer’s instructions (Beyotime, China). Briefly, after culturing for 7 and 14 days, cells were washed three times with PBS and lysed with 1% Triton X‐100 (Beyotime, China) for 30 min on ice. The ALP activity of the supernatants was detected by measuring p‐nitrophenol (p‐NP) and normalized against the total protein content measured by the BCA protein kit. The result was expressed as mM p‐NP produced by each gram of protein (mM p‐NP/gprot). Alizarin red stained calcium nodule were dissolved in 10 mmol/L cetylpyridinium (Coolaber, China) to quantitatively determine matrix calcification, and the absorbance at 562 nm was evaluated with a microplate reader (Thermo Fisher Scientific, USA).

### Adipogenic differentiation

For adipocyte differentiation, 2 days after culturing in complete medium, the medium was replaced with adipogenic medium (1 μM dexamethasone, 0.5 mM isobutylmethylxanthine (Coolaber, China), 0.2 mM indomethacin (Coolaber, China), and 10 mg/mL insulin (Coolaber, China) in complete medium). After another 2 days, the medium was replaced with complete medium containing insulin. The control group was cultured with complete medium. The medium was changed every 2 days. The cells were cultured for 21 days for oil red O (Solarbio, China) staining. The presence of oil droplets was confirmed by staining the cells with 0.3% fresh oil red O solution for 30 min after fixation with 70% ethanol for 10 min. After washing the cells, the stained oil droplets were dissolved with isopropanol and the optical density (OD) of the solution was determined at 520 nm.

### Chondrogenic differentiation

5 × 10^5^ SM-MSCs were transferred into a 15 mL centrifuge tube, centrifuged at 1000 rpm for 5 min, then the supernatant was discarded, and the centrifugation was repeated once to wash the cells with complete medium. The supernatant was discarded and 0.5 mL chondrogenic induction medium (0.1 μM dexamethasone (Sigma, USA), 37.5 μg/mL ascorbic acid (Sigma, USA), 10 ng/mL transforming growth factor-β (TGF-β) (Sigma, USA), 6.25 pg/mL insulin (Coolaber, China), 6.25 μg/mL transferrin (Sigma, USA) in complete medium) was added, while the control group was added complete medium. Centrifuge at 1000 rpm for 5 min, loosen the cap of the centrifuge tube to facilitate gas exchange. When the cells in the induction group formed a pellet, the cartilage balls were released from the bottom of the centrifuge tube by flicking lightly and suspended in the liquid. The induction group was replaced with cartilage induction medium every 3 days, and the cartilage balls were not sucked away when the medium was changed. Control group was replaced with complete medium every 3 days.

After culturing for 21 days, the cartilage pellet was fixed with 4% paraformaldehyde, dehydrated step by step, sectioned with paraffin, stained with Alicin blue (NCM Biotech, China) for 30 min, rinsed with distilled water for 1 min, and observed under the microscope.

### The structure of PRF, the release pattern of growth factors and the preparation of PRF conditioned medium

Venous blood (10 mL) was collected from New Zealand white rabbit's ear margin vein and immediately centrifuged at 3000 rpm for 12 min at room temperature. After squeezing to remove moisture, PRF membranes were prepared.

For observing the ultrastructure of the PRF membrane, the PRF membrane was fixed, dehydrated and sprayed with gold, and then observed under a scanning electron microscope (HITACHI, Japan). The release pattern of growth factors released by PRF was determined by ELISA kit according to the instruction manual (growth factors platelet-derived growth factor (PDGF), TGF-β and vascular endothelial growth factor (VEGF) Elisa Kit, JL Biological, China).

PRF conditional media were prepared as follows (Table 1):

1. α-MEM + PRF: immersed PRF membrane in α-MEM (Hyclone, USA) free of serum and any other components for 14 days and incubated at 37 °C, and then by added 10% FBS, 1%P/S.
2. α-MEM + PRF + osteoinduction: immersed PRF membrane in α-MEM (Hyclone, USA) free of serum and any other components for 14 days, and then by added 10% FBS, 1%P/S, 10 mM β-glycerophosphate, 50 μg/mL ascorbic acid and 10 nM dexamethasone.
3. α-MEM + PRF-non FBS: immersed PRF membrane in α-MEM (Hyclone, USA) free of serum and any other components for 14 days, and then by added 1%P/S.

**Table 1:** A brief description of the components of various conditional media

	α-MEM (Hyclone, USA) and 1%P/S	FBS	PRF	10 mM β-glycerophosphate, 50 μg/mL ascorbic acid and 10 nM dexamethasone
Complete medium (α-MEM)	√	√	×	×
α-MEM + PRF	√	√	√	×
α-MEM + osteoinduction	√	√	×	√
α-MEM + PRF + osteoinduction	√	√	√	√
α-MEM-non FBS	√	×	×	×
α-MEM + PRF-non FBS	√	×	√	×

Other media were (4) complete medium (α-MEM); (5) α-MEM, supplemented with 10% FBS, 1%P/S, 10 mM β-glycerophosphate, 50 μg/mL ascorbic acid and 10 nM dexamethasone (α-MEM + osteoinduction); (6) α-MEM, supplemented with 1%P/S (α-MEM-non FBS). After filtered with a 0.22 μm filter, different medium containing the growth factor of PRF could be obtained. In the culture system, all the culture medium was prepared at the same time to ensure the same serum concentration.

### In vitro assays

α-MEM was added to the E-plate and background impedance was measured. SM-MSCs were collected and seeded on E-plate at a density of 1 × 10^3^/well. After placed in the ultra-clean table at room temperature for 30 min, the E-plate was placed on the test table (the test table was put into the incubator in advance) for real-time and dynamic cell proliferation detection. α-MEM and α-MEM + PRF was added respectively and the real-time dynamic detection was continued to obtain the cell index curve overnight.

The migration of SM-MSCs were evaluated by transwell assay and scratch assay. First, the medium was replaced with serum-free α-MEM to starve SM-MSCs for 12 h. According to the instruction, cells with a density of 1 × 10^4^ in 150 µl of α-MEM-non FBS were seeded into the upper compartment of a transwell chamber with 8 µm pore filters (Corning, USA), and 700 µL of α-MEM-non FBS, (control group) or α-MEM + PRF-non FBS (test group) was added to the lower compartment. After culturing for 12 h, the non-migrated cells were gently wiped off by a cotton swab, and the membranes were fixed in 4% paraformaldehyde for 30 min and stained with crystal violet for 10 min. The number of migrated cells was counted in three randomly selected microscopic fields per well.

For scratch assay, the medium was replaced with serum-free α-MEM to starve SM-MSCs for 12 h. Cells were seeded on 6-well plate at a density of 1 × 10^5^/well and grown until confluence overnight. After scratching a line onto the confluent cell layer, the cells were washed three times with PBS and culture with α-MEM-non FBS and α-MEM + PRF-non FBS. Images of migrating cells were sequentially taken 0, 6, 12 and 24 h after the scratch. The relative scratch region among different sample groups was evaluated and compared.

### Reverse transcription and quantitative polymerase chain reaction

SM-MSCs were seeded at a density of 1 × 10^5^/well in 6-well plates and grown until confluence overnight. After cultured in different media (α-MEM, α-MEM + PRF, α-MEM + osteoinduction, α-MEM + PRF + osteoinduction) for 3, 7 and 14 days, total RNA was extracted using Trizol (TAKARA, Japan) and reverse-transcripted was used TAKARA Reverse Transcriptase kit (TAKARA, Japan) following the manufacturer’s instructions. SYBR®Premix Ex Taq TM (TaKaRa, Japan) was used for real-time PCR with ABI ViiATM7 System (Applied Biosystems, CA) according to the instructions. Data were analyzed by 2^−∆∆Ct^ method and normalized to that of glyceraldehyde-3-phosphate dehydrogenase (GAPDH). All primer sequences of markers are listed in Table 2.

**Table 2:** The primer sequences used for RT-qPCR

Genes	Forward sequence	Reverse sequence
GAPDH	5′-TCACCATCTTCCAGGAGCGA	5′-CACAATGCCGAAGTGGTCGT
Runx2	5′-TCAGGCATGTCCCTCGGTAT	5′-TGGCAGGTAGGTATGGTAGTGG
Col-1	5′-CTTCTGGCCCTGCTGGAAAGGATG	5′-CCCGGATACAGGTTTCGCCAGTA
ALP	5′-CATCTCCCCTCTGGAACTCA	5′-CCAAACAGGAGAGTCGCTTC

### Western blot assay

SM-MSCs were cultured in 6-well plate at a density of 5 × 10^5^/well. After treatment with different media (α-MEM, α-MEM + PRF, α-MEM + osteoinduction, α-MEM + PRF + osteoinduction) for 3 days, total protein was extract from cells using SDS-PAGE Sample Loading Buffer (Beyotime, China). Adjust the sample loading amount according to the protein content of internal reference. Equal amounts of the total protein samples were separated via SDS-PAGE on 10% polyacrylamide gels (Beyotime, China), and the proteins on the SDS gels were transferred onto nitrocellulose membranes (Beyotime, China) and then blocked for 1 h. Incubated with the primary antibodies (anti-ERK 1/2, 1:3000, Abcam, USA; anti-p-ERK 1/2, 1:2000, CST, USA; anti-RUNX2, 1:1000, Santa Cruz, USA; anti-GAPDH, 1:1000, Abbkine, China) overnight at 4 °C. The membranes were washed alternately with PBS and PBST (Beyotime, China). Then the membranes were incubated with the secondary antibodies (AP-labeled IgG (Beyotime, China)) for another 2 h at room temperature. After washed three times and 10 min each time, the signal intensities of the membranes were visualized with the Alkaline Phosphatase Color Development Kit (Beyotime, China). All experiments were performed at least three independent times to obtain consistent results. The relative expression of ERK1/2 and p-ERK1/2 was calculated by the ratio of gray values of the two bands of each protein to the gray values of the internal control band.

### In vivo assay

#### Rabbit maxillary sinus floor elevation model and micro-CT analysis

The processes of exposing the maxillary sinus wall and preparing PRF were described above. Two windows were located about 20 mm anterior to the nasofrontal suture line and 4 mm away from the sagittal suture line. Schneiderian membrane was elevated and the space was filled with equivalent volume of Bio-oss and mixture of Bio-oss and PRF. After 8 and 12 weeks of healing, radiographic examinations were performed for different groups. The present study was approved by the Institutional Animal Care and Use Committee of Jilin University (SY202008003).

#### Bone double-staining

At 6 weeks and 10 weeks of healing, 16 rabbits were intramuscular injected with alizarin red (30 mg/kg, Sigma, USA), and 10 days later, each rabbit was intramuscular injected with calcein (8 mg/kg, Sigma, USA). After 8 and 12 weeks of healing, rabbit maxilla specimens were fixed, dehydrated and resin-cured. Three randomly specimens were sectioned and observed under a laser confocal microscope (Olympus, Japan). The distance between red and green light was analyzed to calculate the rate of bone mineral apposition rate (MAR). The formula is as follows: MAR = the distance between the two fluorescent labeled lines /10 days.

#### Histologic evaluation

The retrieved rabbit maxilla specimens were fixed, decalcified, dehydrated and paraffin embedded. Three randomly specimens were stained with H&E, masson staining and toluidine blue staining. Images were documented under an Inverted microscope (Olympus, Japan).

### Statistical analysis

All quantitative data are presented as the means ± SDs. The continuous data were normally distributed, and the statistical significance was determined using Student's t test or one-way analysis of variance. *p* < 0.05 was accepted as statistically significant. All statistical analyses were conducted with the SPSS 22.0 statistical software (SPSS Inc, USA).

## Results

### Characterization of mesenchymal stem cells derived from SM-MSCs

**Fig. 1:**
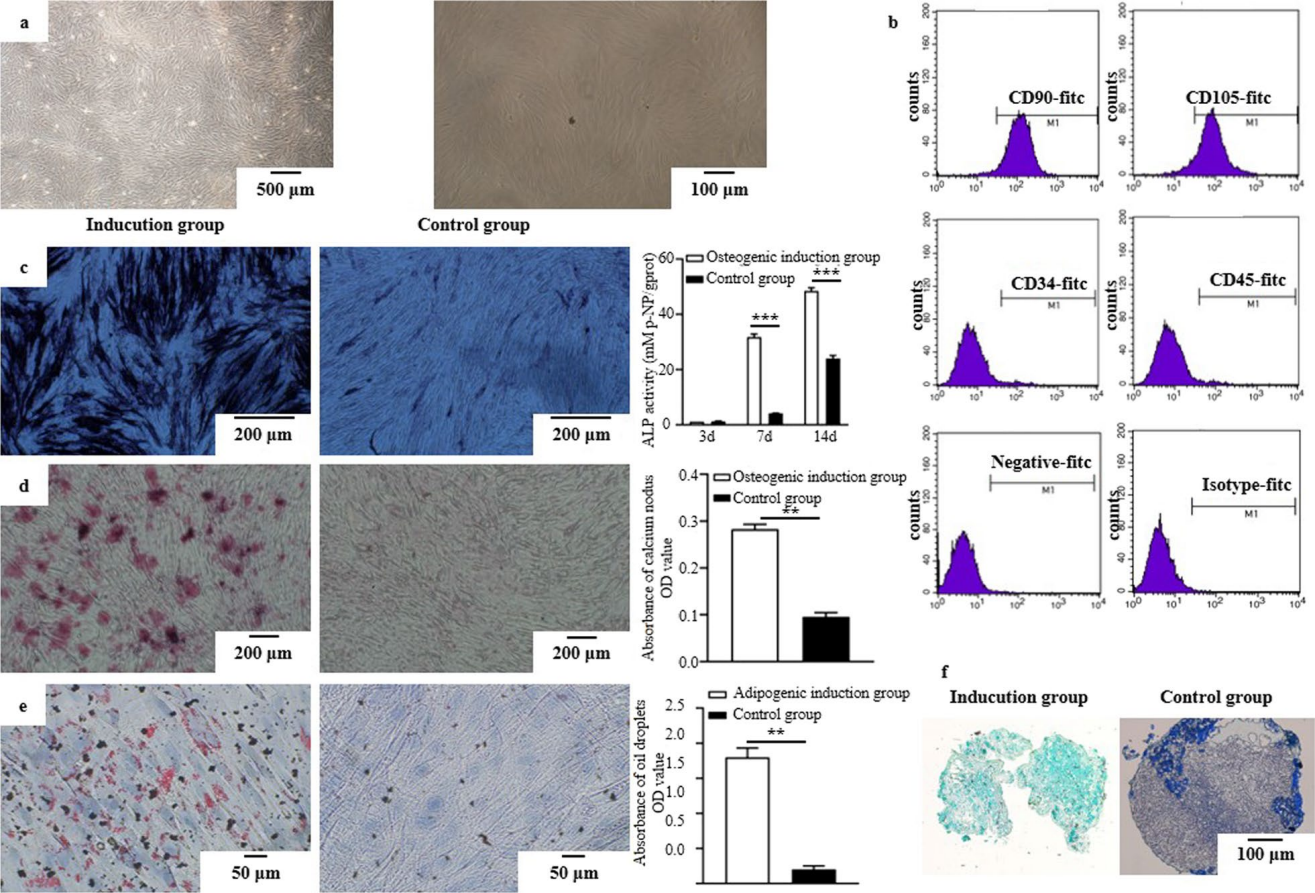
Cells isolated from Schneiderian membrane were identified as MSCs. **a** Cells adhered on plastic dishes as fibroblastic cell‐like morphology with a spindle or star shape. **b** Cells positively expressed the MSC markers CD90 (97.61%) and CD105 (92.71%), but negatively expressed the hematopoietic marker CD34 (2.05%) and the leukocyte marker CD45 (1.46%). **c** Compared with control groups, ALP staining of cells was positive, and ALP activity was significantly enhanced at day 7 and day 14 (*p* < 0.001). **d** Most cells formed mineralized calcium deposits after culturing for 21 days in OM while few mineralized calcium deposits were observed in the control group (*p* < 0.001). **e** Lipid droplets were formed after 21 days of adipogenic induction while there was almost none in the control group (*p* < 0.01). **f** Alicin blue staining showed the blue-stained endoacidic mucopolysaccharides in the pellets in induction group, while less blue staining in control group. **p < 0.01; ***p < 0.001 indicate a significant difference between the groups

The primary SM-MSCs were adhered on plastic dishes as fibroblastic cell‐like morphology with a spindle or star shape (Fig. 1a). After culturing for 10 to 14 days, cells reached confluence. Flow cytometry was carried out to determine cell surface markers. SM-MSCs (P_3_) positively expressed the MSC markers CD90 (97.61%) and CD105 (92.71%), but negatively expressed the hematopoietic marker CD34 (2.05%) and the leukocyte marker CD45 (1.46%), indicating that the isolated cells were mesenchymal lineage (Fig. 1b). To analyze the differentiation potentials of SM-MSCs, osteogenesis, adipogenesis and chondrogenesis inductions were carried out. The osteogenic differentiation of SM-MSCs was verified via ALP staining, ALP activity assay and alizarin red S (ARS) staining. After induction by OM, ALP staining and ALP activity were significantly enhanced compared with control group at day 7 and day 14 (*p* < 0.001) (Fig. 1c). Most cells formed mineralized calcium deposits at 21 days in OM. By contrast, few mineralized calcium deposits were observed in the control group (*p* < 0.01) (Fig. 1d). The adipogenic differentiation capacity of SM-MSCs was verified via oil red O staining. Lipid droplets were accumulated with adipogenic induction for 21 days. These droplets were hardly observed in the control groups (Fig. 1e). Alicin blue staining showed the blue-stained endoacidic mucopolysaccharides in the pellets (Fig. 1f), while there was little blue endoacidic mucopolysaccharide in the control group.

### Ultrastructure and growth factors release pattern of PRF

**Fig. 2:**
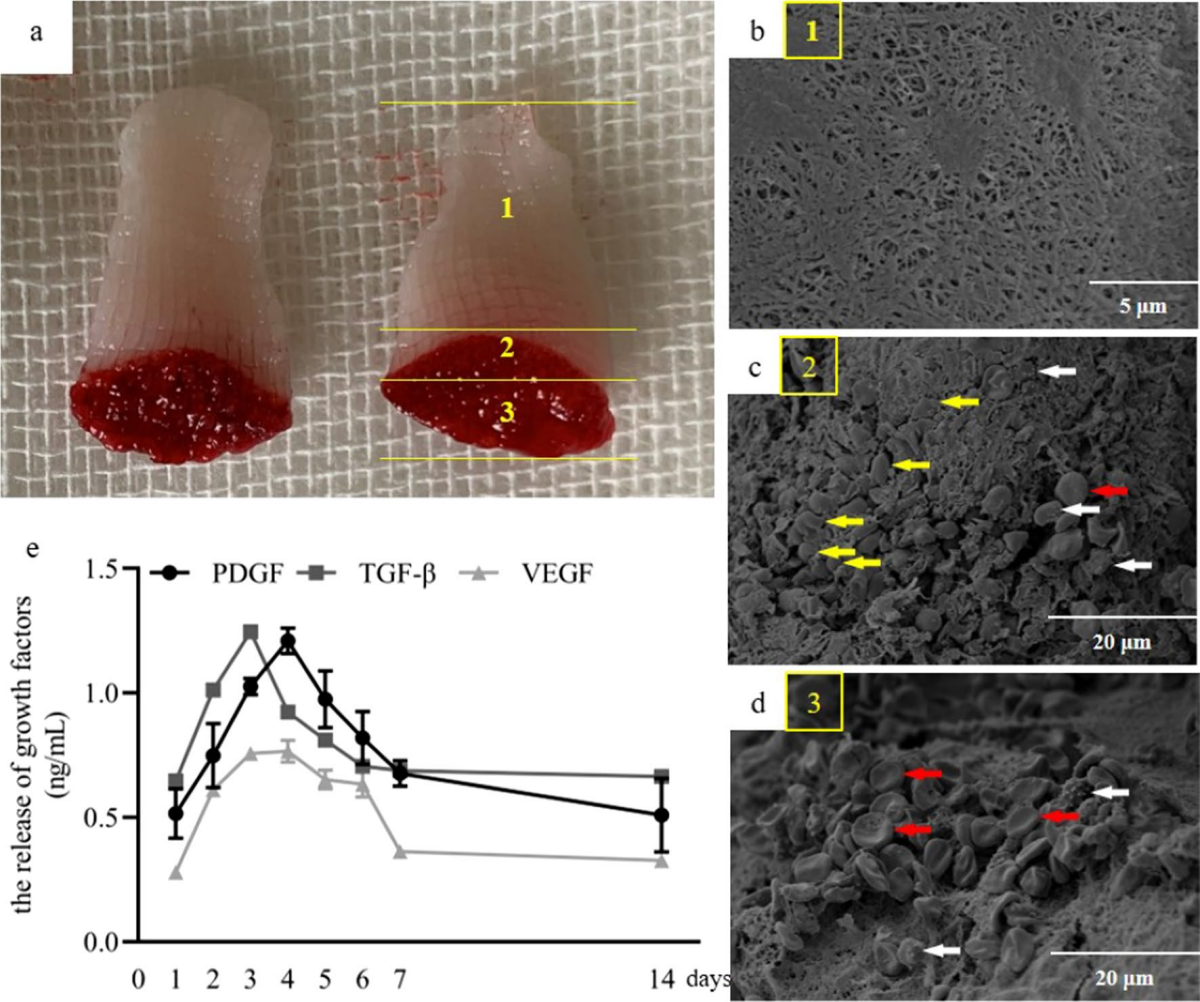
The macrostructure and microstructure of PRF and the release pattern of growth factors. **a** The macrostructure of PRF. **b** In region 1, there was a dense collagen fibrin net with a three-dimensional distribution without cell or platelet attachment. **c** Region 2 was the junction between PRF and erythrocytes. A large number of platelets (yellow arrows), leukocytes (white arrows) and a small number of erythrocytes (red arrows) could be seen. **d** In region 3, a large number of erythrocytes (red arrows) attached to the fibrin net, as well as some platelets (yellow arrows) and leukocytes (white arrows). **e** The release pattern of growth factors was increased first and then decreased

The morphological pattern of the PRF membrane was observed through SEM. The PRF membrane was divided into three parts (Fig. 2a). Region 1 presented a dense collagen fibrin net with a three-dimensional distribution without cells or platelets attachment (Fig. 2b). Region 2 was the junction between the upper yellow region and erythrocytes. Platelets and leukocytes accumulation was observed in the region 2 (Fig. 2c). In region 3, a large number of erythrocytes attached to the fibrin net, as well as a few platelets and leukocytes (Fig. 2d).

As shown in Fig. 2e, the release value of PDGF, TGF-β and VEGF were gradually increased from 1 to 3 days, and presented a sustained release up to 14 days. The maximum value of PDGF was released at day 4. The similar pattern of TGF-β release was observed. The peak release value of TGF-β was observed at day 3. VEGF was released in a stable mass from 3 to 6 days, and began to decrease at day 7.

### The proliferation, migration of SM-MSCs induced by PRF conditioned medium

**Fig. 3:**
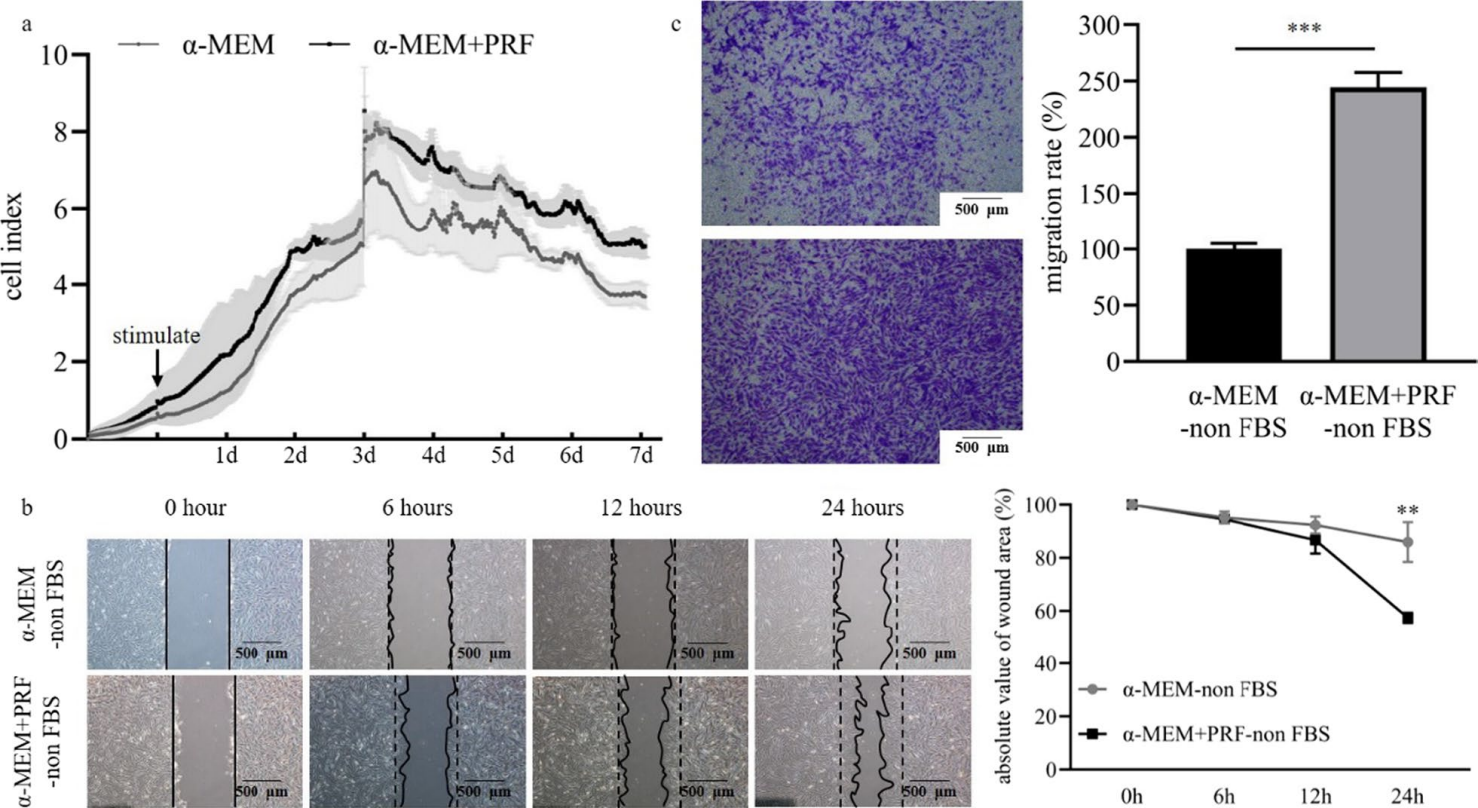
The proliferation and migration of SM-MSCs with or without PRF conditioned medium stimulation. **a** At day 1, the cell index of α-MEM group and α-MEM + PRF group was similar, and from day 2, the cell index of α-MEM + PRF group was higher than that of α-MEM group. **b** Cells began to migrate to the scratch area from 6 h after the scratch formation, and the scratch area in α-MEM + PRF-non FBS group was smaller than that in α-MEM-non FBS group. At 12 h, the scratch area of α-MEM + PRF-non FBS group was significantly smaller than that of α-MEM-non FBS group (*p* < 0.05), the closure rate of the scratch area in the α-MEM + PRF-non FBS group was more than 43% at 24 h, while, the scratch area was closed about 14% in the α-MEM-non FBS group (*p* < 0.05). **c** The diagram of the transwell assay showed that after 12 h, cells migrated to the lower compartment in both groups, but the number of cells migrated in α-MEM + PRF-non-FBS group was significantly higher than that in α-MEM-non-FBS group (*p* < 0.001). ***p < 0.001 indicate a significant difference between the groups

The proliferation of SM-MSCs was first assessed by real-time cell analysis (RTCA) assay, CCK-8 and fluorescence staining. As illustrated in Fig. 3a, the cell activity of α-MEM + PRF group was significantly higher than the α-MEM group up to 7 days. The cell activity was increased from 1 to 3 days, and decreased at day 5. The similar trends were detected by CCK-8 (*p* < 0.05) (Additional file 1: Fig. Appendix 1). Moreover, the detailed cell morphological parameters were displayed by fluorescence staining (Additional file 1: Fig. Appendix 2), the numbers of cells in the α-MEM + PRF group were significantly higher than that in the α-MEM group at day 1, 3, 5 and 7. Therefore, the stimulation of PRF could promote the proliferation of SM-MSCs.

The cell migration was detected by the scratch assay and the transwell assay. The Fig. 3b depicted the cell migration of α-MEM + PRF-non FBS group was statistically faster than the α-MEM-non FBS group. Interestingly, the closure rate of the scratch area in the α-MEM + PRF-non FBS group was more than 43% at 24 h, while, the scratch area was closed about 14% in the α-MEM-non FBS group (*p* < 0.01). The similar trends were shown in the transwell assay. Compared to the control group, PRF significantly induced more than 200% increase in migration at 12 h (*p* < 0.001) (Fig. 3c).

### The osteogenic differentiation of SM-MSCs induced by PRF conditioned medium

**Fig. 4:**
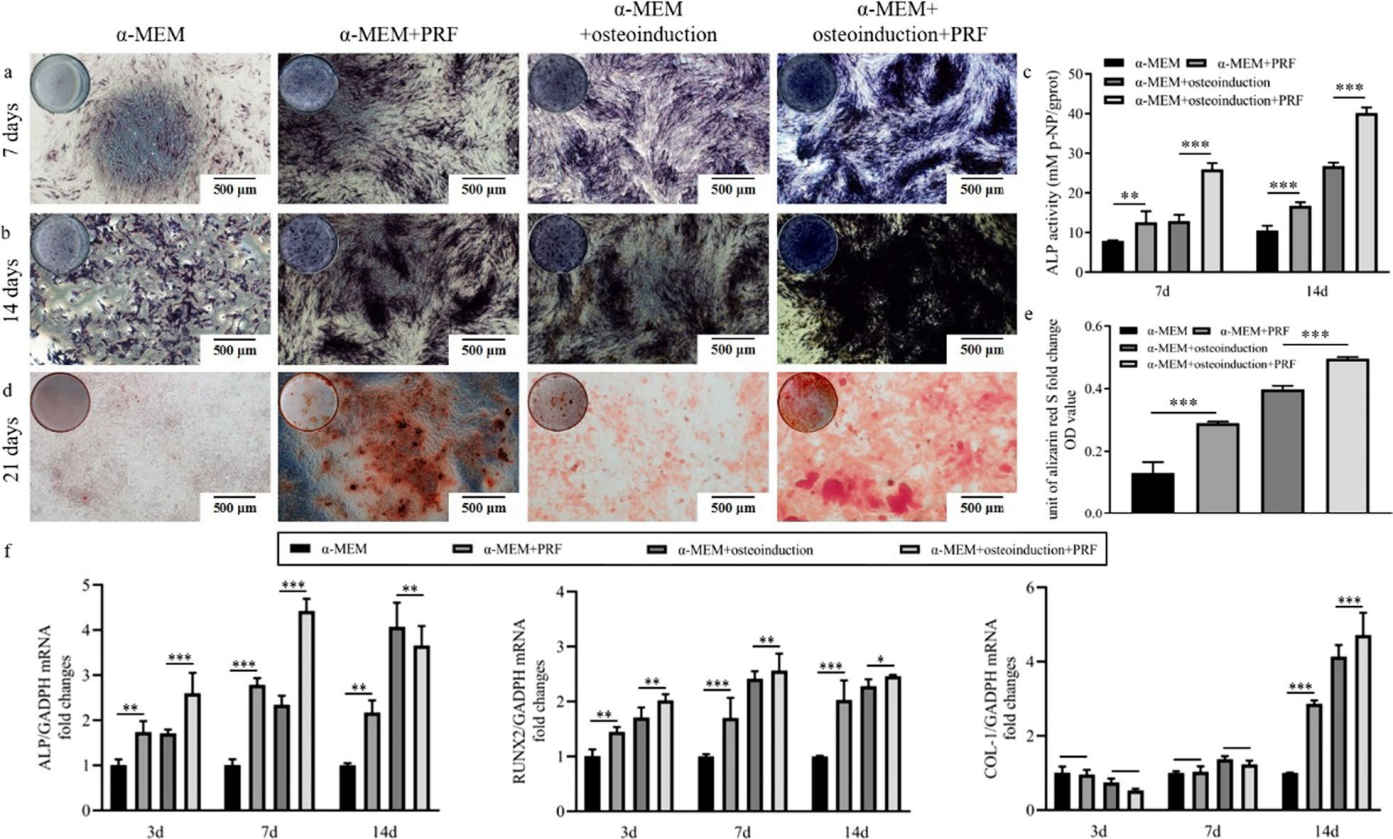
The osteogenic ability of cells with the stimulation of PRF conditioned medium, OM or the combination of above. **a**, **b** The ALP staining results displayed that ALP expression were significantly higher with the PRF presence at 7 days and 14 days whatever the culture conditions. **c** The ALP activity assay results were consistent with the ALP staining results (*p* < 0.01) **d**, **e** There was an increase in positive staining nodules with the PRF presence compared with the corresponding control group. Mineralized nodules dissolution analysis exhibited similar tendency as the Alizarin red staining (*p* < 0.001). **f** the levels of ALP and RUNX2 increased significantly with the PRF presence compared with their corresponding control group (*p* < 0.05) at all experimental time. The similar trends were found in the levels of COL-1 at day 14 (*p* < 0.001). *p < 0.05, **p < 0.01, ***p < 0.001 indicate a significant difference between the groups

To investigate the effects of PRF on the osteogenic differentiation of the SM-MSCs, ALP staining, ALP activity assay, Alizarin red staining and RT-qPCR were performed. As Fig. 4a, b depicted, the ALP staining results displayed that ALP expression were significantly higher with the PRF presence at 7 days and 14 days whatever the culture conditions. The ALP activity assay results were consistent with the ALP staining results (*p* < 0.01) (Fig. 4c). Notably, the ALP staining of the α-MEM + PRF group was similar with the α-MEM + osteoinduction group, indicating that PRF had similar osteogenic induction effect to SM-MSCs compared with OM. As Fig. 4d exhibited, there was an increase in positive staining nodules with the PRF presence compared with the corresponding control group. Mineralized nodules dissolution analysis exhibited similar tendency as the Alizarin red staining (*p* < 0.001) (Fig. 4e).

The expression levels of ALP, RUNX2 and COL-1 were detected by RT-qPCR as shown in Fig. 4f, showing that the levels of ALP and RUNX2 were increased significantly in the other three groups compared with α-MEM group at 3, 7 and 14 days (p < 0.05). Moreover, the levels of ALP and RUNX2 were increased significantly with the PRF presence compared with their corresponding control group (p < 0.05) at all experimental time. While the expression of COL-1 was decreased compared with their corresponding control group at day 3 and 7, however, there was no significant difference, while increased at day 14 (p < 0.000).

### Regulation of ERK 1/2 signaling pathway on PRF conditioned medium to promote osteogenic differentiation of SM-MSCs

**Fig. 5:**
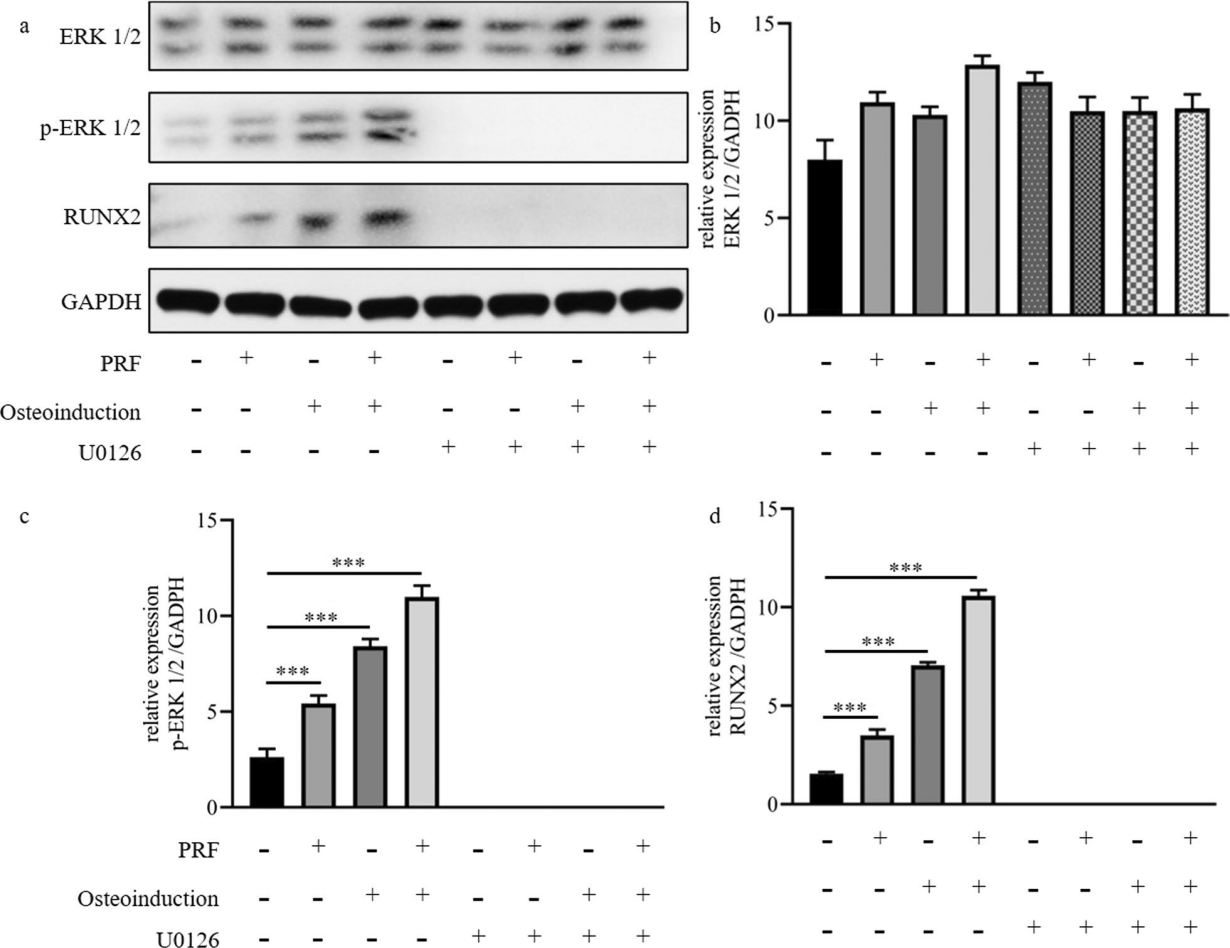
The protein expression levels of p-ERK 1/2 and Runx2 were notably up-regulated without the ERK 1/2 signaling pathway inhibitor U0126 compared the α-MEM group (*p* < 0.001), especially, the α-MEM + osteoinduction + PRF group. However, the protein expression levels of p-ERK 1/2 were remarkably down-regulated with the ERK 1/2 signaling pathway inhibitor U0126. Even more, the protein of Runx2 were not expressed with the ERK 1/2 signaling pathway inhibitor U0126. ***p < 0.001 indicate a significant difference between the groups

Western blot analysis was performed to further investigate the osteogenic mechanism. Figure 5 depicted that the protein expression levels of p-ERK 1/2 and Runx2 were notably up-regulated without the ERK 1/2 signaling pathway inhibitor U0126 compared the α-MEM group (*p* < 0.001), especially, the α-MEM + osteoinduction + PRF group. However, the protein expression levels of p-ERK 1/2 were remarkably down-regulated with the ERK 1/2 signaling pathway inhibitor U0126. Even more, the protein of Runx2 were not expressed with the ERK 1/2 signaling pathway inhibitor U0126.

### Enhancement of PRF in bone augmentation of maxillary sinus floor elevation

**Fig. 6:**
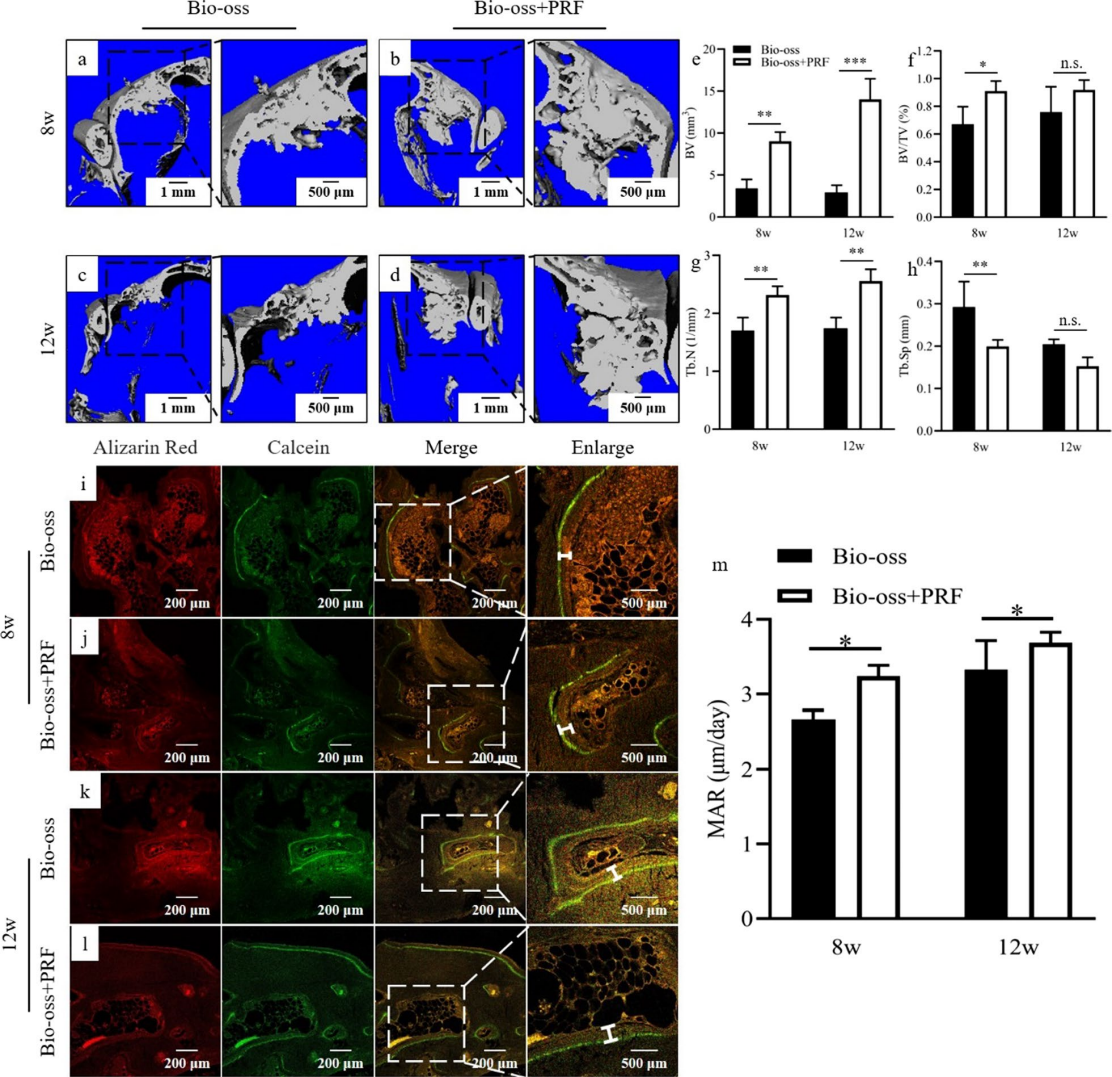
In vivo results. **a**, **b** At 8 weeks post-surgery, 3D reconstruction was performed in the Bio-oss group and the Bio-oss + PRF group. **c**, **d** At 12 weeks post-surgery, 3D reconstruction was performed in the Bio-oss group and the Bio-oss + PRF group. **e** Bone volume (BV) of Bio-oss + PRF group was significantly higher than Bio-oss group at 8 and 12 weeks post-surgery (*p* < 0.01). **f** Bone volume fraction (BV/TV) of Bio-oss + PRF group was higher than Bio-oss group at 8 and 12 weeks post-surgery, but the difference was statistically significant only at 8 week (*p* < 0.05). **g** Number of bone trabeculae (Tb.N) of Bio-oss + PRF group was significantly higher than Bio-oss group at 8 and 12 weeks post-surgery (*p* < 0.01). **h** Trabeculae separation (Tb.Sp) of Bio-oss group was higher than Bio-oss + PRF group at 8 and 12 weeks post-surgery, but the difference was statistically significant only at 8 weeks (*p* < 0.01). **i**, **j** At 8 weeks post-surgery, alizarin red and calcein were labeled in the Bio-oss group and the Bio-oss + PRF group. **k**, **l** At 12 weeks post-surgery, alizarin red and calcein were labeled in the Bio-oss group and the Bio-oss + PRF group. **m** MAR of Bio-oss + PRF group was significantly higher than that in Bio-oss group, and the difference was statistically significant (*p* < 0.05). *p < 0.05; **p < 0.01; ***p < 0.001 indicate a significant difference between the groups

The micro-CT was performed to observe new bone formation at 8 and 12 weeks post-surgery. The 8 and 12 weeks post-surgery micro-CT imaging were shown as Fig. 6a–d. The new maxillary sinus wall was formed, and a mass of newly formed bone was observed in the maxillary sinus. Significant higher bone volum (BV) value was noted for Bio-oss + PRF group than that in Bio-oss group at 8 and 12 weeks post-surgery (*p* < 0.05) (Fig. 6e). The bone volume fraction (BV/TV) in the Bio-oss + PRF group was significantly higher than that in the Bio-oss group at 8 weeks post-surgery (*p* < 0.05), while there was no significantly different at 12 weeks post-surgery (Fig. 6f). The number of bone trabeculae (Tb.N) showed the similar trends as the BV value (*p* < 0.01) (Fig. 6g). Moreover, the trabeculae separation (Tb.Sp) in the Bio-oss + PRF group was significantly lower than that in the Bio-oss group at 8 weeks post-surgery (*p* < 0.05) (Fig. 6h).

To further evaluate new bone formation, bone double-staining with alizarin red and calcein were performed. Representative images of each group were shown in Fig. 6i–l. The bone tissue was marked red and green under different wavelength of excited light. And the distance which was between the red and green label represented the bone mineral apposition during 10 days. At 8 and 12 weeks post-surgery, MAR in the Bio-oss group was markedly slower than the Bio-oss + PRF group (*p* < 0.05) (Fig. 6m), indicating that the application of PRF could accelerate the formation of new bone in the early stage of osteogenesis. Immunofluorescence assay showed that ERK 1/2 was expressed in both of Bio-oss and Bio-oss + PRF group, however, the expression of RUNX2 in Bio-oss group was lower than that in Bio-oss + PRF group, which was consistent with the in vitro experiments (Additional file 1: Fig. Appendix 3).

**Fig. 7:**
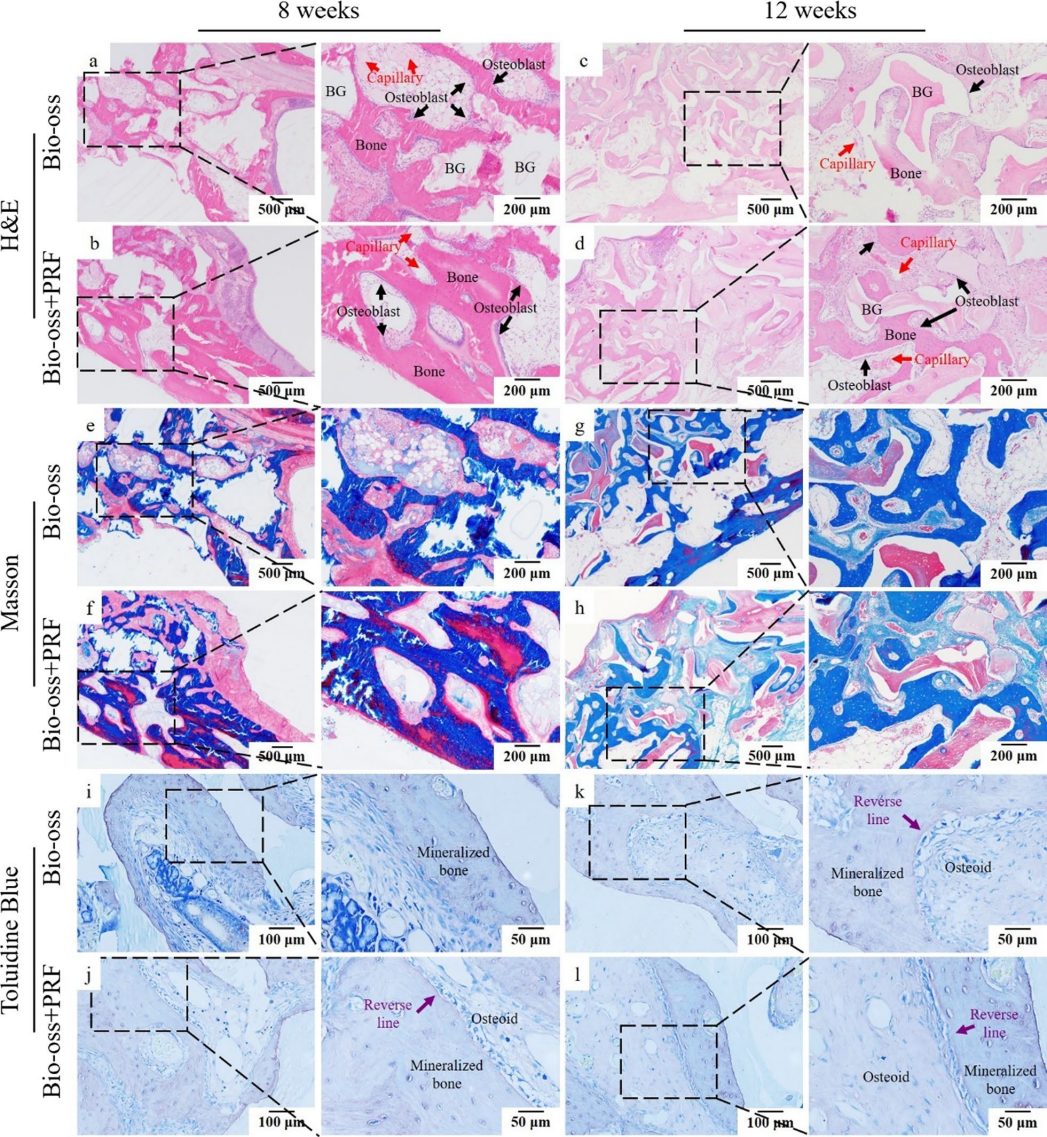
Histological observation of Bio-oss + PRF group and Bio-oss group. **a**–**d** In 8 and 12 weeks, compared with the Bio-oss group, the bone in the Bio-oss + PRF group was more mature, with less Bio-oss granules (BG), less osteoid, and more capillaries (red arrow) and active osteoblasts (black arrow). **e**–**h** In 8 and 12 weeks, compared with the Bio-oss group, there was more collagen fiber and mature bone tissue in Bio-oss + PRF group. **i**–**l** In 8 and 12 weeks, compared with the Bio-oss group, the number of osteoid and Bio-oss particles was less and mineralized bone was more in Bio-oss + PRF group. Purple reverse linecould be seen between the newly formed bone and osteoid

The regenerated bone was stained red by HE staining. At 8 weeks post-surgery, sparse newly formed bone was found in the Bio-oss group, a large number of Bio-oss particles were present in the bone graft area, and the woven bone was formed in the early stage. There were active osteoblasts arranged in rows between the mature bone tissue and the woven bone tissue. The woven bone was filled with a large number of capillary vessels, and the osteogenic activity was active (Fig. 7a). In the Bio-oss + PRF group, there were a small amount of non-osteogenic Bio-oss particles, less woven bone and reticular bone, and more mature bone tissue. Moreover, a large number of active osteoblasts and abundant capillaries were present around the mature bone tissues (Fig. 7b). Over time, 12 weeks post-surgery, a large number of Bio-oss particles and large areas of immature reticular bone were still visible in the Bio-oss group, and osteoblasts were arranged between woven bone and mature lamellar bone (Fig. 7c). In the Bio-oss + PRF group, the Bio-oss particles were less than those in the Bio-oss group. The grafts were mostly substituted by mature lamellar bone, with active osteoblasts arranged in rows between the mature bone and osteoid (Fig. 7d). The quantitative of mature bone area (Additional file 1: Fig. Appendix 4a *p* < 0.05) statistically reconfirmed the aforementioned results.

The masson staining showed collagen fibers as blue (Fig. 7e–h). In the Bio-oss + PRF group, the blue staining area was larger than the Bio-oss group, indicating more collagen fibers and a higher maturity of bone tissue at 8 weeks after surgery. At 12 weeks post-surgery, collagen fibers in the Bio-oss group gradually increased, but large areas of non-fibrous woven bone were still visible. The area of collagen fibers in the Bio-oss + PRF group was further expanded, and some lightly stained areas could be seen, indicating the gradual maturation of bone tissue and the accumulation of collagen fibers. The percentage of collagen area verified the above graphical results. (Additional file 1: Fig. Appendix 4b, *p* < 0.01). The red and blue staining near the wall of the maxillary sinus indicates that the newly formed collagen fibers were more mature near the wall of maxillary sinus than that near Schneiderian membrane (Fig. 7f, h).

The toluidine blue staining showed newly formed bone as blue (Fig. 7i–l). At 8 and 12 weeks post-surgery, toluidine blue staining showed a large number of bone with different maturity. There were more osteoid and Bio-oss particles in the 8 weeks post-surgery than that in the 12 weeks post-surgery. At the same time point, compared with the Bio-oss group, the bone tissue of the Bio-oss + PRF group was more mature, and there were more mineralized and deposited mature bone. There was a purple line between the newly formed bone and the mineralized bone.

## Discussion

Nowadays, MSFE has been considered as the most effective procedure for bone augmentation in the posterior maxilla. Osteogenic potential of SM-MSCs was confirmed, involving in the new bone formation during the MSFE procedure [22]. Many growth factors, such as BMP-2, PDGF, TGF-β and insulin like growth factor-1 (IGF-1), were considered as the potent regulators of bone formation [23]. PRF, as the autograft, contains abundant growth factors and platelets. In our previous study, we confirmed the use of PRF alone could obtain a certain amount of new bone formation during MSFE procedure [19]. This present study was conducted to further explored the PRF efficacy on the biological behaviors and its possible mechanism of SM-MSCs. In this study, MSCs from Schneiderian membrane were successfully isolated and identified. PRF conditioned medium promotes the proliferation, migration and osteogenic differentiation of SM-MSCs due to its abundant growth factors. PRF conditioned medium or OM, or the combination of above two could promoted osteogenic differentiation by upregulating ERK 1/2 signaling pathway. The animal experiments showed that the application of PRF could significantly accelerate the rate of new bone formation in the maxillary sinus and increase the quantity and quality of newly formed bone. However, it is undeniable that the new bone near the wall of maxillary sinus was more mature, while the bone formation near the Schneider membrane was less mature.

The International Society for Cell and Gene Therapy defines the term "MSCs" and establishes minimum criteria for defining MSCs [24]: (1) cells grow adherently on plastic dishes; (2) Cell surface markers CD105, CD73, and CD90 were positively expressed, while CD45, CD34, CD14, CD11b, CD79a, CD19, and HLA-I were negatively expressed; (3) Cells can be induced to differentiate to at least three directions, such as bone, cartilage, fat, and nerve. Currently, SM-MSCs have been isolated from human [10] and pig [7], and were positive for CD105, CD146, CD166, CD71, CD73 and other MSC markers, and negative for CD34 [25]. Osteogenic differentiation showed that SM-MSCs could be induced to differentiate towards osteogenesis and express the osteogenic genes RUNX2 and OCN [26]. For chondrogenic and adipogenic differentiation, Guo et al. reported that SM-MSCs could be differentiated into adipocytes and chondrocytes. In the present study, SM-MSCs were successfully isolated from Schneiderian membrane. Cells positively expressed CD 90 and CD105 and negatively expressed CD 34 and CD 45, and had multidirectional differentiation potential. The results obtained in present study met the criteria for the identification of MSCs, indicating SM-MSCs had the multi-lineage differentiation potential of MSC.

With the deepening of research on autologous platelet concentrate, PRF, as is an autologous platelet concentrate that is rich in platelets and leukocytes [15], has been widely used in clinical practice [27]. Activated platelet contained in PRF could release a large number of growth factors such as PDGF, VEGF, TGF-β, IGF [28, 29]. In in vivo or clinical study, due to its abundant growth factors, PRF could be used as graft material and had excellent ability in promoting bone regeneration in extraction socket [30], repairing fracture [31] and improving gingival retreat [32]. However, studies on the growth factors release regularity of PRF in vivo are rare, since growth factors also exist in large quantities in human or animal bodies, it is not clear whether the detected growth factors were contained in the body itself or were released from PRF. Some scholars reported that compared with the non-graft group, the growth factor in the gingival sulcus increased at day 3 after PRF was grafted into the periodontal defect and remained at a higher level until day 14 [33]. However, the results above could only provide some indirect evidence for the growth factors release regularity of PRF in vivo. As reported in a large number of studies, growth factors such as PDGF, TGF-β, VEGF, BMP could be released continuously from PRF in vitro up to 14 days [33–35], which was consistent with our results. In this study, the structure of PRF was observed, and it was found that leukocytes and platelets were abundant at the junction between PRF layer and erythrocyte layer, and PRF could slowly release the growth factors of PDGF, VEGF and TGF-β. Among those growth factors, PDGF is a potent connective tissue cell growth factor that binds to specific high-affinity receptors expressed on a variety of cell surfaces and is a powerful chemical attractant and stimulator of cell proliferation [23]. It was confirmed that all subtypes of PDGF could promote the proliferation of periodontal ligament stem cells (PDLSCs) in vitro and stimulate the synthesis of collagen [36] and hyaluronic acid [37]. PDGF promotes bone formation by affecting cell proliferation, migration, differentiation and extracellular matrix synthesis [38]. VEGF is the most important factor responsible for angiogenic cell differentiation and angiogenesis. It is the most effective stimulator of endothelial cell proliferation, migration and angiogenesis. It stimulates osteogenesis by regulating osteogenic growth factor through paracrine signals and improves vascular permeability [39]. VEGF can promote bone regeneration, such as femoral fracture in mice and radial segment defect in rabbits, which can achieve good bone regeneration [40]. The TGF-β family is one of the most important superfamilies of factors regulating growth and differentiation [41]. Under physiological conditions, TGF-β1 regulates the recruitment of endogenous MSCs to bone remodeling sites and regulates osteoblastic differentiation [42]. Although TGF-β1 promotes the recruitment and proliferation of MSCs and induces them to differentiate into early osteoprogenitor cells, several studies have shown that TGF-β inhibit the maturation, mineralization, and osteocyte transition of MSCs through the ALK5/Smad3 pathway [43]. Therefore, TGF-β is a multifactorial regulator of multidirectional regulation of cell growth and development, and its regulatory role is very complex. In the study, PRF conditioned medium can promote SM-MSCs proliferation, migration and osteogenic differentiation, attributing to the presence of the abundant of growth factors in the PRF. In addition, PRF application promoted the expression of ALP, RUNX2, and COL-1 in the present study. The expression of these three transcription factors is closely related to osteogenic differentiation. Knocking out Runx2 gene resulted in a decrease in osteoblasts required for bone remodeling but an increase in the bone marrow adiposity. It was strongly concluded that Runx2 is essential for differentiation of MSCs to the osteoblastic lineage [44]. ALP plays a key role in early osteogenesis and hydrolyzes various types of phosphate to promote osteoblastic maturation and calcification [45]. COL-1 is a marker of late osteogenic differentiation. The unmineralized collagen fibers in the mineralized matrix continue to mineralize as the minerals secreted by osteoblasts are deposited [46]. The upregulation of the expression of these transcription factors implies that the application of PRF conditioned medium promotes osteogenic differentiation of SM-MSC, involving in the bone formation.

A large number of signaling pathways and cytokines constitute a complex network and interact each other, thus influencing the osteogenic differentiation of MSCs. In addition to Wnt /β-catenin, Notch and other signaling pathways, ERK 1/2 signaling pathway is widely expressed and is an important link between the cell surface and nucleus to regulate proliferation and differentiation migration, and cell death [47]. Besides, the ERK 1/2 signaling pathway plays a critical role in bone formation, since it can crosstalk with all molecular pathways. Xiao et al. reported that activation of α2β1 integrins by collagen I matrix is involved in stimulating osteoblast differentiation through activation of the ERK 1/2 signaling pathway [48]. In addition, these clavicle and skull phenotypes were corrected when Runx2^+/−^ mice were crossed with MEK1 transgenic mice to constitute active mutants, directly demonstrating the relationship between Runx2 and the ERK signaling pathway. Shi et al*.* confirmed that MSCs overexpression of p-ERK 1/2 could significantly increase the expression level of Runx2 [49]. In another study, PRF could enhance the phosphorylation of ERK in PDLSC and increase the expression of ALP and osteoprotegerin in cells [50]. In this study, the application of PRF conditioned medium up-regulated the expression of p-ERK1/2, and thus up-regulated the expression of Runx2, indicating that PRF promoted osteogenic differentiation of SM-MSCs through up-regulation of ERK 1/2 signaling pathway.

Based on our results that PRF exhibited strongly osteogenic stimulative effects on SM-MSCs in vitro, we further evaluated its effects on bone regeneration during MSFE in vivo. In present study, it is not hard to find that active osteoblasts could be found either on the side of the Schneiderian membrane or on the side of parietal wall of the maxillary sinus, and new bone was formed on either side through histological observation. The osteogenic pattern is not only from the wall of maxillary sinus to the Schneiderian membrane, but also from the Schneiderian membrane to the wall of maxillary sinus. Undeniably, the quality and maturity of the new bone near the wall of maxillary sinus were better than that of the new bone near Schneiderian membrane. It is unknown that whether the osteoblasts forming new bone on the side of the Schneiderian membrane originate from the maxillary sinus wall or from the Schneiderian membrane. But there is no doubt that MSCs can be isolated from Schneiderian membrane in vitro. The osteogenic potential of Schneiderian membrane plays a certain role in the internal osteogenesis of maxillary sinus. Growth factors released by PRF may play an important osteoinductive role in the formation of mature bone and dense bone trabeculae [51] and shown an excellent effect on the rate and amount of new bone formation [52].

## Conclusion

The present study confirmed the existence of MSCs in Schneiderian membrane, and PRF could promote cells proliferation, migration and osteogenic differentiation. PRF contributes to the formation of new bone in maxillary sinus. However, the unknown origin of osteoblasts forming new bone in maxillary sinus in vivo is a limitation of this study. Further localization analysis can be carried out to determine the origin of osteogenesis.

## Supplementary Information

**Additional file 1.** Supplementary File.

## Data Availability

All data generated or analyzed during this study are included in this published article and its supplementary information files.

## Abbreviations

MSC: Mesenchymal stem cell
PRF: Platelet-rich fibrin
SM-MSC: Schneiderian membrane derived mesenchymal stem cell
OM: Osteoinductive medium
ALP: Alkaline phosphatase
PCR: Polymerase chain reaction
MSFE: Maxillary sinus floor elevation
BMP: Bone morphogenetic protein
FBS: Fetal bovine serum
P/S: Penicillin and streptomycin
p-NP: p‐nitrophenol
OD: Optical density
ARS: Alizarin red staining
PDGF: Growth factors platelet-derived growth factor
VEGF: Vascular endothelial growth factor
TGF-β: Transforming growth factor-β
RTCA: Real-time cell analysis
COL-1: Collegen I
IGF-1: Insulin like growth factor-1
BV: Bone volume
BV/TV: Bone volume fraction
Tb.Sp: Number of bone trabeculae
Tb.N: Trabeculae separation
MAR: Mineral apposition rate
OCN: Osteocalcin
PDLSC: Periodontal ligament stem cells
