# Supplementary material for: Effects of platelet-rich fibrin on osteogenic differentiation of Schneiderian membrane derived mesenchymal stem cells and bone formation in maxillary sinus
Source: Cell Commun Signal. 2022 Jun 15;20:88. doi: 10.1186/s12964-022-00844-0 (PMC9202141; doi:10.1186/s12964-022-00844-0)
Supplement: Supplementary file 2 — Additional file 1. Supplementary File. [file 12964_2022_844_MOESM2_ESM.docx]

**Materials and methods**

**CCK-8 assay**

Cells were seeded in 96-well plates at a density of 1 × 10^3^/well and were cultured overnight in a cell incubator. After 24 h, fresh complete medium was replaced in the α-MEM group, and α-MEM+PRF group was replaced with α-MEM+PRF medium. The medium was changed every 3 days. After culturing for 1, 3, 5, 7 days, 10 μl CCK-8 was added to each well. The plate was shaken to mix well, and the cells were incubated in a cell incubator for 1 h. The absorbance was measured at 450 nm wavelength.

**Cell fluorescence staining**

For showing cell proliferation status more visually, cells were seeded in 24-well plates at a density of 1 × 10^4^/well. Cells were fixed in 4% paraformaldehyde in for 30 min after culturing for 1, 3, 5, 7 days in α-MEM or α-MEM+PRF and incubated with FITC (Sigma, USA) and DAPI (Sigma, USA) respectively dying [cytoplasm](javascript:;) and nucleus. Cell density was observed by inverted fluorescent microscopy (Olympus, Japan) on different days.

**Immunofluorescence assay**

Samples were fixed with 4% paraformaldehyde in PBS overnight at 4 ℃, followed by decalcification in EDTA for 3 months, dehydration with a graded sucrose solutions (15% and 30% sucrose for 2 hours each at room temperature, and 30% sucrose with 50% OCT overnight at 4 ℃) and immediately embedded in OCT (Sakura Finetek, 4583). Fresh Frozen tissue blocks were sectioned at 8 µm on a cryostat (Leica) and mounted on SuperFrost Plus slides (Fisher), and staining immediately. Before staining, samples were drying under 60℃ for over 3h, then wash 3 times in PBS, 5 min for each. Sections were permeabilized with 0.5-1% TritonX-100/PBS for 10 min and then washed 3 times in PBS, 5 min for each. Sections were blocked with blocking buffer containing 1% BSA, 2% goat serum and 0.3% TritonX-100 in PBS for 1 h at room temperature. The samples were incubated with the primary antibodies overnight at 4 ℃: anti-ERK 1/2 (1:3000, Abcam, USA); anti-RUNX2 (1:1000, Santa Cruz, USA)/blocking buffer containing 1% BSA, 2% goat serum, with the ratio of 1:1000-1:2000. 8. Then wash 5 times in PBST, 5 min for each. Wash 5 times in PBS, 5 min for each. Then the sections were incubated with fluorescently conjugated secondary antibodies: Alexa Fluor (Invitrogen)/blocking buffer containing 1% BSA, 2% goat serum, with the ratio of 1:500-1:1000. Wash 5 times in PBST, 5 min for each. Then wash 5 times in PBS, 5 min for each. Then Images were captured immediately.

**Results**

The CCK-8 results showed cell activity increased first and began to decrease at day 5. Compared with α-MEM group, the cell activity of α-MEM+PRF group was higher, and the difference was statistically significant (*p*<0.05) (Fig Appendix 1). Moreover, the detailed cell morphological parameters were displayed by fluorescence staining (Fig Appendix 2), the numbers of cells in the α-MEM+PRF group were significantly more than that in the α-MEM group at day 1, 3, 5 and 7.


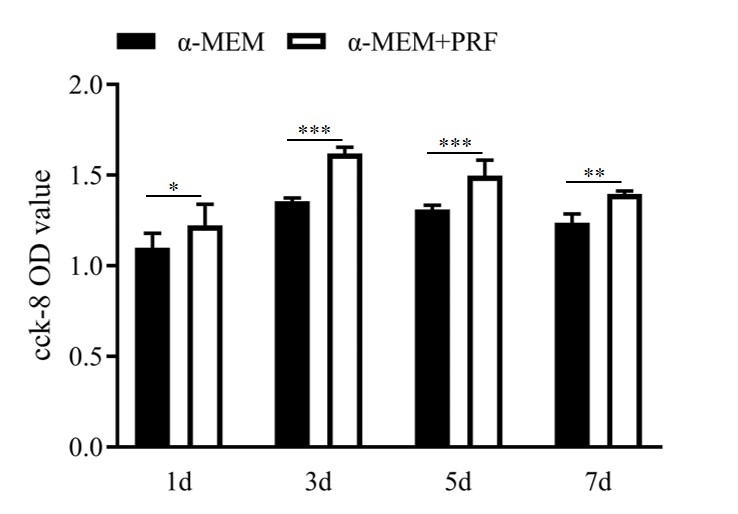


Fig Appendix 1. Cell activity increased first and then decreased. Compared with α-MEM group, the cell activity of α-MEM+PRF group was higher, and the difference was statistically significant (*p*<0.05). * *p*<0.05, ***p*<0.01, ****p*<0.001 indicate a significant difference between the groups.


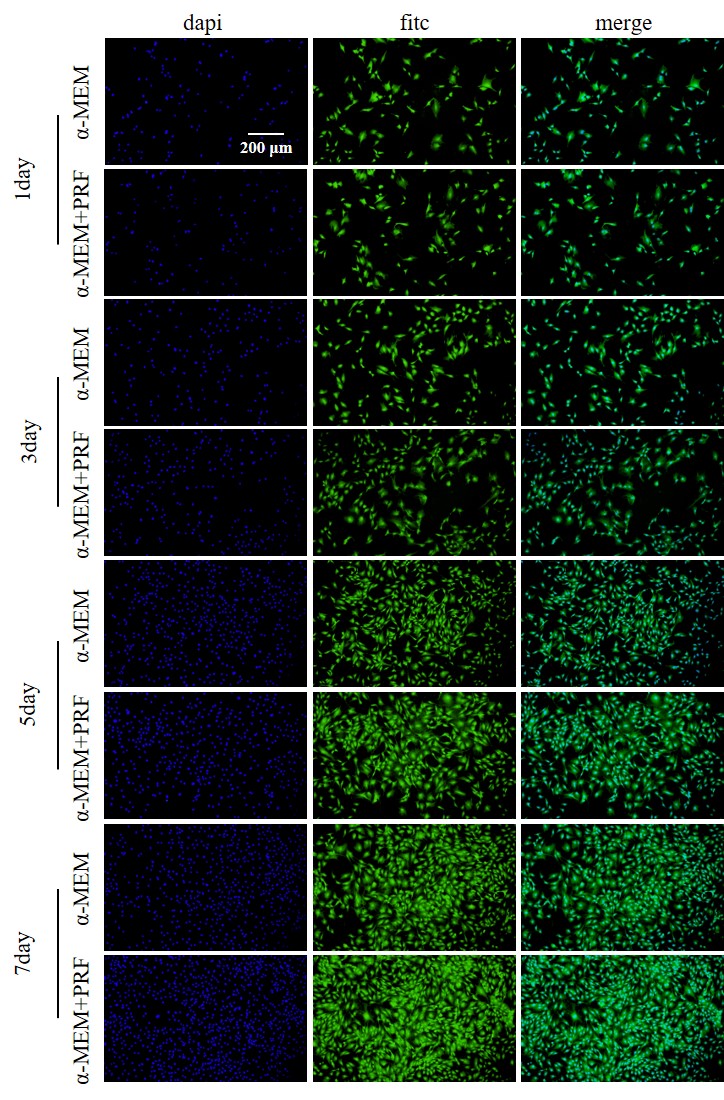


Fig Appendix 2. The numbers of cells in the α-MEM+PRF group were significantly more than that in the α-MEM group at 1, 3, 5 and 7 days.

Immunofluorescence assay showed that ERK 1/2 was expressed in both of Bio-oss and Bio-oss+PRF group, however, the expression of RUNX2 in Bio-oss group was lower than that in Bio-oss+PRF group (Fig Appendix 3), which was consistent with the *in vitro* experiments.


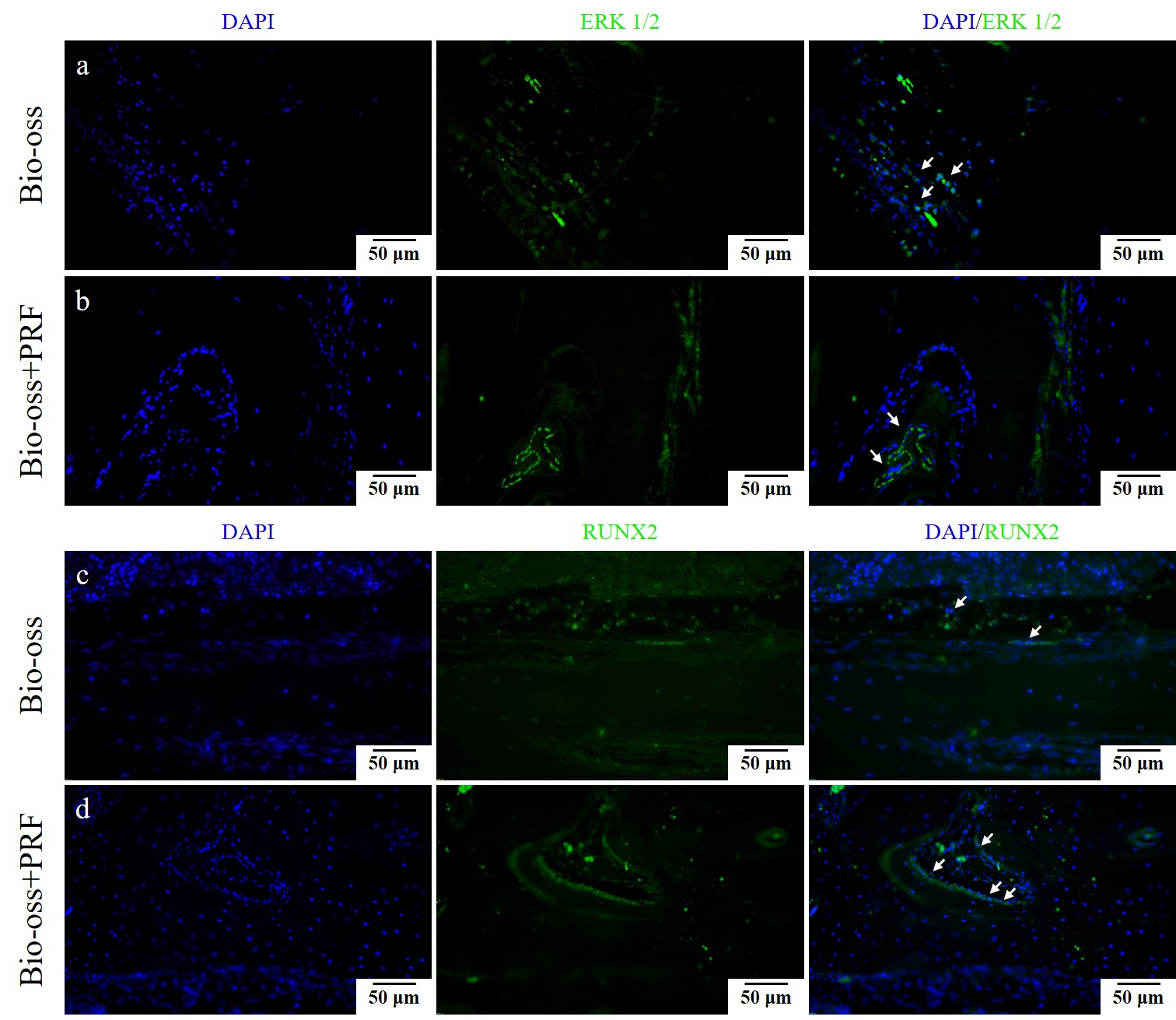


**Fig Appendix 3.** The immunofluorescence images of ERK1/2 and RUNX2 in vivo. **a, b**. ERK 1/2 expressed in Bio-oss and Bio-oss+PRF group. c, d. RUNX2 expressed in Bio-oss and Bio-oss+PRF group.

The masson staining showed collagen fibers as blue (Fig 7e-h, Fig Appendix 4). In the Bio-oss+PRF group, the blue staining area was larger than the Bio-oss group, indicating a higher maturity of bone tissue at 8 weeks after surgery (p<0.01).


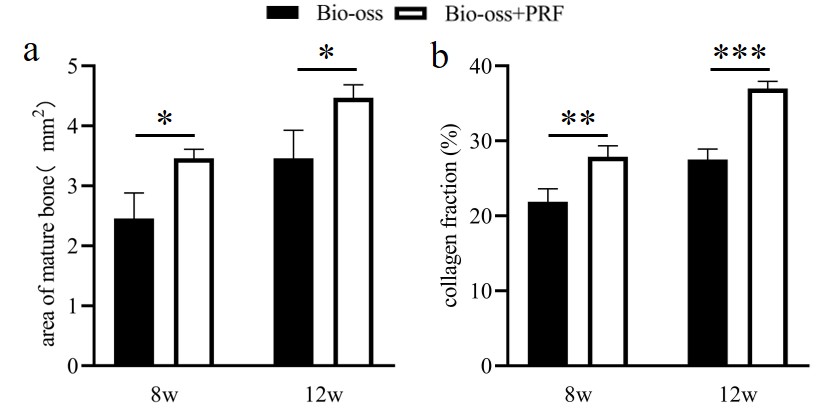


Fig Appendix 4. Area analysis of mature bone and collagen. a: The areas of mature bone were significantly different between Bio-oss group and Bio-oss+PRF group both in 8 weeks (*p*=0.41) and 12 weeks(*p*=0.47). b: The percentage of collagen area was indicated by the collagen fraction. **p*<0.05, ***p*<0.01, ****p*<0.001 indicate a significant difference between the groups.
